# 187 A Systematic Review of Dance Programs on Health and Wellbeing of Children and Adolescents

**DOI:** 10.1093/eurpub/ckae114.146

**Published:** 2024-09-26

**Authors:** Fergal O’Connor, Siobhán O’Reilly, Amanda Clifford, Patrick Brosnan, Aoife Whiston

**Affiliations:** School of Allied Health, University of Limerick, Limerick, Ireland; School of Allied Health, University of Limerick, Limerick, Ireland; Ageing Research Centre, Health Research Institute, University of Limerick, Limerick, Ireland; School of Allied Health, University of Limerick, Limerick, Ireland; Ageing Research Centre, Health Research Institute, University of Limerick, Limerick, Ireland

## Abstract

**Background:**

Physical inactivity is a prevalent issue in youths, with less than 25% of children achieving the recommended 60 minutes of physical activity daily. Dance is a form of physical activity that can improve physical and mental wellbeing within different populations. The primary aim of this review was to explore the effectiveness of dance as a form of physical activity for children and adolescents.

**Methods:**

A structured search strategy was conducted across seven databases to identify randomised controlled trials that investigated the effectiveness of dance programs on school-going children and adolescents. Studies published from January 2003 to December 2023 were included. Data regarding study characteristics including participants, dance interventions, and outcomes of interest were extracted and narratively synthesised. A meta-analysis was performed on outcomes where possible.

**Results:**

Seventeen studies met the inclusion criteria, the majority of which were of good quality and had a low risk of bias based on the Physiotherapy Evidence Database (PEDro) scale and Risk of Bias 2 tools respectively. Despite most studies reporting positive effects, following meta-analysis, no significant improvements were found on working memory or non-executive cognitive function, with high levels of heterogeneity observed for both groups. Negative effects on non-executive cognitive function were reported by one study which incorporated a short dance intervention (10 minutes per week x 9 weeks) In general, the effect of dance interventions on physical activity and on physiological characteristics were equivocal showing either no effect, slight improvements, or significant improvements. These mixed results indicated that dance had similar effects as regular physical education (PE) classes but showed slight improvements over educational lessons and no intervention.

**Conclusion:**

Of the studies included, there were mixed results regarding the effect of dance interventions on cognitive, physical activity, and physiological outcomes for children and adolescents. The studies that used a suitable intervention duration reported no negative effects which suggests that dance can be used as a safe exercise modality and provide similar benefits to regular PE lessons. Further research should include larger sample sizes, interventions tailored towards the intended outcome, and use established core outcome sets to facilitate easier comparability across trials.

